# Early emergence increases survival of tree seedlings in Central European temperate forests despite severe late frost

**DOI:** 10.1002/ece3.5399

**Published:** 2019-07-03

**Authors:** Eva Bianchi, Harald Bugmann, Christof Bigler

**Affiliations:** ^1^ Forest Ecology, Department of Environmental Systems Science, Institute of Terrestrial Ecosystems ETH Zurich Zurich Switzerland

**Keywords:** *Acer*, *Fagus*, frost, mortality, seed germination, seedling emergence, survival time, tree regeneration

## Abstract

Global warming is expected to result in earlier emergence of tree seedlings that may experience higher damages and mortality due to late frost in spring. We monitored emergence, characteristics, and survival of seedlings across ten tree species in temperate mixed deciduous forests of Central Europe over one and a half year. We tested whether the timing of emergence represents a trade‐off for seedling survival between minimizing frost risk and maximizing the length of the growing period. Almost two‐thirds of the seedlings died during the first growing period. The timing of emergence was decisive for seedling survival. Although seedlings that emerged early faced a severe late frost event, they benefited from a longer growing period resulting in increased overall survival. Larger seedling height and higher number of leaves positively influenced survival. Seedlings growing on moss had higher survival compared to mineral soil, litter, or herbaceous vegetation. *Synthesis*. Our findings demonstrate the importance of emergence time for survival of tree seedlings, with early‐emerging seedlings more likely surviving the first growing period.

## 1 INTRODUCTION

Global temperatures are rising concurrent with an increase in the magnitude and frequency of extreme weather events (Intergovernmental Panel on Climate Change, 2014). Higher mean temperatures typically accelerate biological processes such as stem elongation or leaf unfolding in plants, or development of seedlings (Matías & Jump, 2014; Saxe, Cannell, Johnsen, Ryan, & Vourlitis, 2001). However, these processes respond also to weather extremes, not only to means (Fisichelli, Vor, & Ammer, 2014; Inouye, 2000). For example, damage in unfolding leaves of plants is likely to occur in response to frost events even under global warming (Cannell & Smith, 1986; Rigby & Porporato, 2008). Leaf damages are typically observed when late frost events with freezing temperatures below a critical threshold occur during or shortly after leaf unfolding (Bigler & Bugmann, 2018; Charrier, Ngao, Saudreau, & Améglio, 2015; Guillaume, Isabelle, Marc, & Thierry, 2018; Vitasse, Lenz, & Körner, 2014).

Similar to adult trees, tree seedlings are most susceptible to frost during the unfolding of cotyledons and primary leaves, with older foliar tissue becoming less susceptible (Sakai & Larcher, 1987). The frost resistance of seedlings differs across species (Hofmann, Jager, & Bruelheide, 2014), that is, mortality may vary in response to late frost events, because seedlings of different species have different survival strategies and react differently to environmental influences (e.g., frost, drought, shading, and substrate) due to different ecophysiological traits. As frost damage in spring may be lethal for seedlings, the timing of emergence is expected to affect survival. Premature or delayed timing of seedling emergence is suboptimal in mixed deciduous forests. On the one hand, seedlings with early emergence benefit from (a) a higher rate of photosynthesis related to higher light availability on the forest floor, since in deciduous forests emergence occurs before canopy closure and (b) a longer growing period, which means improved carbohydrate reserves, larger seedling size, more leaves, and thus higher survival (Dunlap & Barnett, 1983; Jones, Allen, & Sharitz, 1997; Seiwa, 1998; Seiwa, Ando, Imaji, Tomita, & Kanou, 2009; Urbieta, Pérez‐Ramos, Zavala, Marañón, & Kobe, 2008). However, seedlings with an early emergence may suffer from environmental hazards early in the season (Larson & Funk, 2016) such as late frost events (Shimono & Kudo, 2003). On the other hand, delayed germination decreases frost risk but usually occurs under ongoing or completed canopy closure and also reduces the length of the growing period, which may result in a reduced size of the root system, smaller seedling size, fewer leaves, and insufficient carbohydrate reserves to survive drought events (Leverett, Schieder, & Donohue, 2018). Thus, the timing of emergence and the risk of late frost events represent a crucial trade‐off for seedling survival.

The importance of emergence time for survival of annual and perennial plants (e.g., trees) is well known from numerous studies (Battaglia, 1996; Gioria, Pyšek, & Osborne, 2016; Miller, 1987; Verdú & Traveset, 2005). However, the combination of the effect of emergence time with the effects of late frost and drought on seedling survival has not been systematically investigated to date, in spite of its large significance in the face of global climate change. In addition, previous studies have not assessed the joint effects of emergence time and time‐varying factors (e.g., changes in radiation, temperature, precipitation, or seedling characteristics) on survival within seedling age cohorts under natural conditions (McNair, Sunkara, & Frobish, 2012). Most studies have focussed on biotic and abiotic effects on seedling mortality at a certain point in time (e.g., at the end of the growing period), often from seeds sown under experimental conditions (Dulamsuren, Hauck, & Leuschner, 2013; Frei et al., 2018; Hunziker & Brang, 2005), but without considering shorter observation intervals (Larocque, Shugart, Xi, & Holm, 2015) and natural regeneration under natural conditions, respectively. Moreover, the seedbed and microsites on which emergence and growth occur were found to influence seedling survival (Berkowitz, Canham, & Kelly, 1995), but their role has rarely been assessed at the small spatial scale of individual seedlings.

In our study, we observed seedling emergence from seeds naturally present in temperate mixed deciduous forests of Central Europe under field conditions. We monitored seedling survival of the 2017 cohort over one and a half years, starting in April, which was an exceptionally warm spring followed by a severe late frost (Vitasse & Rebetez, 2018). We addressed the following research questions: (a) Does the timing of emergence influence tree seedling survival? (b) Does survival over the first growing period vary among tree species? (c) Does seedling survival differ across species in response to biotic variables (substrate and seedling characteristics such as height and number of leaves) and abiotic variables (frost, warmth, drought and light availability)?

## 2 MATERIALS AND METHODS

### 2.1 Study design and surveys of seedlings

Our study encompasses ten tree species of Central European temperate mixed deciduous forests: *Acer pseudoplatanus* L. (sycamore), *Fagus sylvatica* L. (beech), *Fraxinus excelsior* L. (ash), *Abies alba* Mill. (silver fir), *Carpinus betulus* L. (common hornbeam), *Picea abies* (L.) H. Karst (Norway spruce), *Acer platanoides* L. (Norway maple), *Pinus sylvestris* L. (Scots pine), *Quercus robur* L. (common oak), and *Tilia cordata* Mill. (small‐leaved lime). We focussed the analyses on the first four species, which have different ecophysiological traits, with shade tolerance being very high in beech and silver fir, and high in sycamore and ash; drought tolerance being moderate in silver fir, sycamore and ash, and low in beech; and frost resistance being moderate in sycamore and very low in beech, silver fir and ash (Leuschner & Meier, 2018; Matter & Schütz, 2002).

In March 2017, three sites were established on the hills Üetliberg, Hönggerberg, and Zürichberg in the vicinity of the city of Zürich, Switzerland. The Üetliberg site is located at 700 m a.s.l., the Hönggerberg site at 520 m a.s.l., and the Zürichberg site at 600 m a. s. l. The center of each site is located at an intersection point of the 1 km ×1 km grid of the Swiss reference coordinate system, corresponding to the WG84 coordinates 47°21’37’’N 8°29’04’’E (Üetliberg), 47°24’51’’N 8°29’56’’E (Hönggerberg), and 47°23’44’N 8°33’53’’E (Zürichberg). Distances from center to center are 6.1 km between Üetliberg and Hönggerberg, 7.3 km between Üetliberg and Zürichberg, and 5.4 km between Hönggerberg and Zürichberg. Within a square area of 200 m × 200 m around each center, 25 intersection points on a 50 m × 50 m grid were potential plot locations. To avoid disturbance of the plots, close proximity to frequently used forest trails was a criterion for exclusion of seven intersection points at each of the three sites. Plots of 1 m × 2 m were set up at the remaining 18 intersection points.

In these 54 plots (3 sites × 18 plots per site), natural emergence, characteristics, and survival of tree seedlings were assessed approximately biweekly from the beginning of April 2017 (i.e., the first sampling occurred prior to the severe frost in mid‐April) to the end of October 2017 by counting and individually marking them in each plot. The survival of the seedlings, but not the emergence of new seedlings, was assessed again in two surveys after the first winter, that is, in mid‐April 2018 and after the second growing period in mid‐October 2018. Survival of the first growing period was defined as the percentage of emerged seedlings surviving until the end of October 2017. Winter survival was defined as the percentage of seedlings surviving the period from early November 2017 to mid‐April 2018, whereas survival of the second growing period was defined as the percentage of seedlings surviving the period from mid‐April 2018 to mid‐October 2018. Seedling height was measured with a hand ruler perpendicularly from the soil surface to the apical meristem. The number of cotyledons and euphylls was assessed at each survey. If present and attributable, cause and severity of seedling damages were recorded. We assessed the extent of browsing damage by estimating the missing foliar tissue, but we could not discern the animal species that caused the browsing. In June 2017, we estimated for each seedling within a radius of 3 cm (hereafter called “seedbed”) and for each plot (hereafter “microsite”) the percentage of the surface covered by moss, mineral soil, litter, and herbaceous vegetation (including grasses and forbs).

Daily values of mean, minimum, and maximum air temperatures at 5 cm above ground as well as daily precipitation sums were measured at the MeteoSwiss climate stations Affoltern (located approx. 2.1 km from the site) for Hönggerberg, Fluntern (1.9 km) for Zürichberg, and Waldegg (precipitation only, 2.1 km) for Üetliberg.

At each plot, one temperature logger (iButton; Maxim Integrated Products, Sunnyvale, CA, USA) that was shielded from radiation was used to record air temperature at a height of 10 cm every 30 min. At each site, two temperature loggers further recorded soil temperature at a depth of 5 cm every 2 hr. The height and depth chosen for the loggers are biologically relevant for aboveground growth of germinants through the growing period, and for soil freezing in the shallow rooting zone of the germinants, respectively. Soil volumetric water content (HydroSense; Campbell Scientific, Logan, UT, USA) was measured at every survey with 12‐cm long rod probes in six equally distributed points across each plot. Canopy hemispherical photographs were taken at every survey in the north‐western corner of each plot. We used a Canon EOS 70D camera with a Sigma 4.5 mm F2.8 Model EX DC HSM circular fisheye lens (Sigma Corporation of America, Ronkonkoma, NY, USA). The camera base was mounted at 40 cm above ground. Hemispherical photographs were analyzed with the software Hemisfer, version 2.2 (Schleppi, Conedera, Sedivy, & Thimonier, 2007) to calculate the daily effective diffuse and direct radiation available at each plot. At the Hönggerberg site, two plots were destroyed during the measurement campaign and these seedlings were thus not used in the analyses.

### 2.2 Statistical analyses

Statistical analyses were performed mainly with data of the first growing period, that is, data of the winter and of the second growing period were analyzed only descriptively because these last two surveys (each with a six‐month observation interval) were temporally too distant compared to those during the first growing period (each with approximately a two‐week observation interval). Our survival data were subject to both truncation (Kleinbaum & Klein, 2012) and censoring (Klein & Moeschberger, 2003), because they were (a) left‐ and right‐truncated (i.e., we did consider neither seedlings that emerged and died before the beginning of the study nor seedlings that emerged after the end of the study, respectively); (b) left‐ and right‐censored (i.e., seedlings emerged already before the beginning of the study, although we assume mid‐March 2017 as earliest possible emergence time, and seedling surviving beyond the last survey, respectively); and (c) interval‐censored (i.e., emergence and death times were only known to have occurred within the interval between two surveys). We therefore handled survival times as doubly‐interval‐censored data, with minimal and maximal differences between emergence and death times.

To study the development of survival over time, we adopted two approaches. The first was based on the survival function *S*(*t*), that is, the probability of surviving up to time *t*:

(1) S(t)=pr(T>t),0<t<∞

where *T* is survival time (Moore, 2016). We used the Kaplan–Meier method (Kaplan & Meier, 1958) to estimate survival curves. The estimate of a survival curve visualizes the survival probability, which takes value 1 at time 0, as a nonincreasing step function over time. A survival probability of 0.5 corresponds to the median survival time of a group of seedlings. We grouped seedlings by seedbed, microsite, species, site, and month of emergence, respectively.

The second approach was based on the hazard function *h*(*t*), that is, the instantaneous failure rate at time *t*:

(2) $$h\left(t\right)=\lim_{\delta \to 0}\frac{\text{pr}(t<T<t+\delta \left|T\right.>t)}{\delta }$$

where *h*(*t*) corresponds to the probability that a seedling surviving up to time t dies in the next small interval of time, divided by the length of that interval (Moore, 2016). The survival function *S*(*t*) can be expressed as a negative exponential cumulative hazard function *H*(*t*):

(3) $$S\left(t\right)=\exp\left(-\int_{0}^{t}h\left(u\right)du\right)=\exp\left(-H\left(t\right)\right)$$

Based on this relationship between survival and hazard, it is possible to compare survival curves among groups without assuming a particular form of the underlying survival distribution. Hazard differences between groups were tested using the proportional hazards assumption:

(4) $$h_{1}\left(t\right)=\psi h_{0}\left(t\right)$$

where *h*_0_(*t*) is the baseline hazard and $\psi =e^{z\beta }$ or $\log\left(\psi \right)=z\beta$. The parameter β is the log‐hazard ratio for the effect of a given variable *z* on survival (i.e., a negative β indicates a reduced hazard and thus an increased survival, whereas a positive β indicates an increased hazard and thus a decreased survival). The proportional hazards assumption along with the above‐mentioned censoring and nondefined survival distribution are the fundamentals of Cox's proportional hazards model (Cox, 1972), which relies on partial likelihood (“Cox models” below).

With the second approach based on the hazard function *h*(*t*), we conducted two types of survival analysis. In the first part, we used Cox models to compare survival curves among groups (levels of a factor), that is, to statistically test for differences within a single time‐constant covariate (Martinussen & Scheike, 2006). We fitted five Cox models, each with one single fixed effect using the function *coxph* of the *survival* package, version 2.41‐3 (Therneau, 2015) in the statistical computing software *R* (R Core Team, 2017), version 3.4.3. For the two models that we used to test for seedbed and microsite effects, moss was set as baseline hazard (i.e., reference level for the other surface cover types). The model to test for species differences used beech (*Fagus sylvatica* L.) as baseline hazard since this species experienced the highest mortality (see section Results) and is the dominant species in most forests of the Swiss Plateau. In the model testing for site differences, Zürichberg was the baseline hazard since this site had the lowest total emergence and the highest mortality. Finally, the model testing for differences between months of emergence, which defined the levels of a factor, had the level March as the baseline hazard. Unlike the second part of the survival analysis, the data in the first part cannot be considered completely independent, because no random effects could be used, and therefore the true *p*‐values are larger than estimated.

To statistically assess the relationship of explanatory variables to survival time (Crowder, 2012), in the second part of the survival analysis we used Cox models with Gaussian random effects, also known as frailty models or mixed‐effects Cox proportional hazards models (Therneau & Grambsch, 2000; Wienke, 2010). We modelled survival with covariates including random effects and time‐dependent covariates. We fitted four mixed‐effects Cox models, each for one of the four most common tree species (i.e., species with >200 emergences in the entire study) using the function *coxme* of the *coxme* package, version 2.2‐7 7 (Therneau, 2018) in the software *R* (R Core Team, 2017). We fitted species‐specific models because the species have distinct ecological characteristics such as the timing of emergence, and their survival may be influenced in a different way by various factors. We used random effects, with seedlings nested within plots and plots nested within sites, in order to take into account the heterogeneity among individuals (Moore, 2016) as well as the clustering and spatial proximity of seedlings. Our seedling are independent observations, as in spite of being in the same plot they differed with respect to species, emergence time, and further variables (e.g., exposure to frost, height, number of leaves, substrate). In contrast to the previously fitted Cox model that considered month of emergence as a factor, emergence time was included in the mixed‐effects Cox models as a continuous variable, that is, the day of the year when a seedling was recorded for the first time. Two groups of time‐dependent, continuous covariates were included as fixed effects: seedling‐specific covariates (number of cotyledons, number of euphylls, and seedling height) and abiotic covariates (negative minimum temperatures, positive maximum temperatures, mean precipitation sum, and mean direct radiation). Negative minimum temperature was used to estimate the effects of frost on survival of tree seedlings, positive maximum temperature, and mean precipitation sum represented drought effects. Time‐dependent covariates are particularly suitable when covariates feature strong fluctuations over time (Fisher & Lin, 1999), such as air temperature or the number of euphylls during the course of the growing period.

## 3 RESULTS

### 3.1 Weather

**Figure 1:**
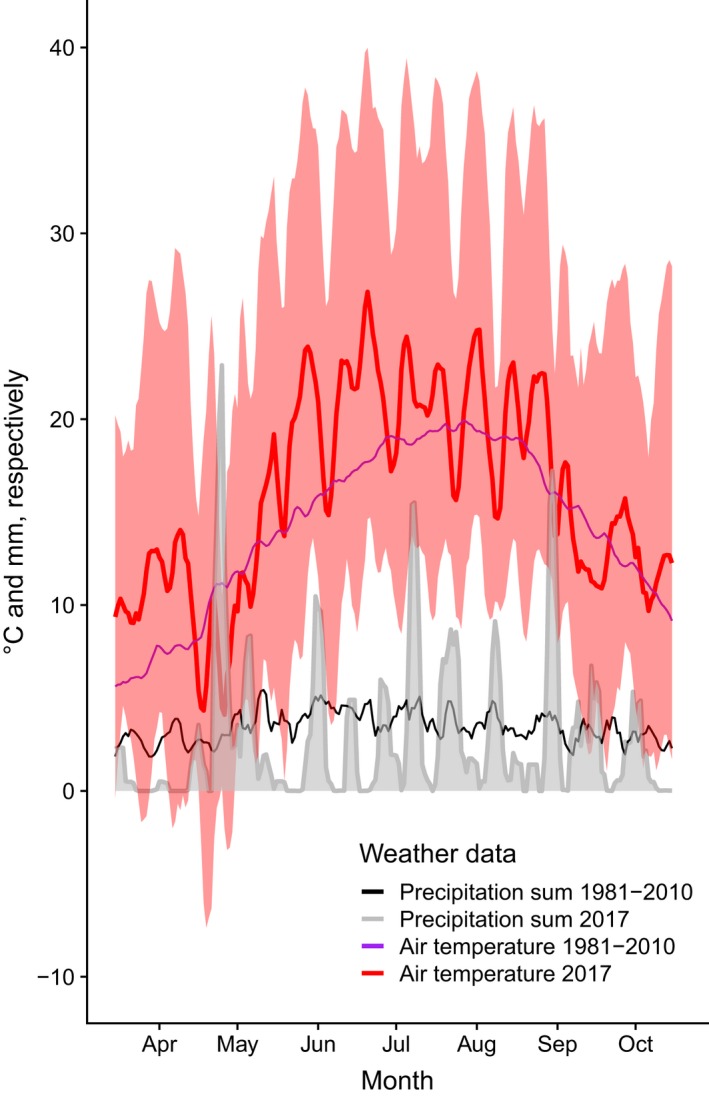
Weather data of MeteoSwiss ground level monitoring networks of the growing period in Zürich for the long‐term average 1981–2010 and for the 2017, with running averages of 4 days. Mean, minimum, and maximum daily air temperatures at 5 cm above grass recorded at the MeteoSwiss climate stations Üetliberg, Affoltern and Fluntern (purple and red). Daily precipitation sum recorded at the MeteoSwiss climate stations Waldegg, Affoltern and Fluntern (black and gray). Source MeteoSwiss

Both spring and summer 2017 (Figure 1) were the third warmest seasons on record in Zürich since the beginning of the measurements in 1864, with a mean air temperature of 1.7°C and 1.9°C above the long‐term average of 1981–2010, respectively (MeteoSwiss, 2018). March 2017 featured the second warmest temperatures (3.3°C above average), inducing one of the earliest spring vegetation developments of the last 30 years, which was evident in the leaf unfolding of common forest tree species (MeteoSwiss, 2018). Following this unusually warm period, severe late frost events occurred from 19 to 22 April 2017 with minimum air temperatures reaching −3°C at 10 cm above ground logged in our plots and unusually late snowfall observed on 26 and 28 April 2017. In May and June 2017, precipitation in Zürich amounted to only 62% and 67% of the average precipitation sum of 122 mm and 128 mm, respectively, compared to the long‐term average of 1981– 2010, that is, late spring/early summer was exceptionally dry (MeteoSwiss, 2018). Following this period of drought, heat spells intermittently occurred from June to August 2017 (MeteoSwiss, 2018). Air temperatures logged at 10 cm height above ground were consistently lowest at the Üetliberg site and highest at the Zürichberg site compared to the other sites, whereas soil temperatures at 5 cm depth were lowest at the Üetliberg site and similar at the other two sites (Table 1).

**Table 1:** Monthly mean air and soil temperatures (mean ± 1 *SD*) for the three sites from April 2017 to March 2018

Year	Month	Hönggerberg site [°C]	Üetliberg site [°C]	Zürichberg site [°C]
Air temperature				
2017	April	8.9 ± 3.7	7.9 ± 4.0	8.8 ± 4.1
	May	14.1 ± 4.2	13.3 ± 4.5	14.0 ± 4.4
	June	18.8 ± 3.2	18.0 ± 3.4	18.7 ± 3.3
	July	18.2 ± 2.5	17.4 ± 2.8	18.0 ± 2.7
	August	18.5 ± 3.0	17.7 ± 3.2	18.3 ± 3.0
	September	12.6 ± 2.1	11.5 ± 2.1	12.1 ± 2.1
	October	10.6 ± 2.6	10.1 ± 3.0	10.4 ± 2.7
	November	4.3 ± 2.8	3.6 ± 3.3	4.2 ± 3.0
	December	0.9 ± 1.8	−0.1 ± 1.9	0.6 ± 1.8
2018	January	4.2 ± 1.9	3.5 ± 2.0	4.0 ± 2.0
	February	−1.0 ± 2.8	−2.5 ± 3.2	−1.6 ± 3.1
	March	3.5 ± 3.6	2.5 ± 4.0	3.1 ± 3.8
Soil temperature				
2017	April	9.3 ± 2.8	7.0 ± 2.1	8.9 ± 1.8
	May	12.7 ± 3.0	10.6 ± 3.0	11.7 ± 2.3
	June	17.0 ± 2.1	15.4 ± 1.9	16.1 ± 1.6
	July	17.2 ± 1.6	15.8 ± 1.4	16.9 ± 1.2
	August	17.5 ± 1.9	16.3 ± 1.8	17.7 ± 2.1
	September	12.9 ± 1.6	11.8 ± 1.5	13.1 ± 1.7
	October	11.5 ± 1.4	10.6 ± 1.6	11.9 ± 1.6
	November	6.3 ± 1.9	5.0 ± 2.0	7.3 ± 1.6
	December	2.4 ± 0.8	1.7 ± 0.7	3.8 ± 0.7
2018	January	4.5 ± 1.3	3.7 ± 1.2	5.2 ± 1.0
	February	1.2 ± 1.6	0.4 ± 1.5	2.4 ± 1.4
	March	3.2 ± 2.7	1.7 ± 1.8	3.3 ± 2.0

### 3.2 Changes in light availability

Light availability varied among plots and decreased in the course of the growing season with ongoing canopy development, as canopy closure started in mid‐April 2017. The leaf unfolding of shrubs and herbaceous plants at the forest floor, for example, *Allium ursinum* L. occurring in some plots at the Üetliberg site, took place after mid‐April 2017. Thus, early‐emerging seedlings experienced high light availability and minor understory competition.

### 3.3 Seedling emergence

**Figure 2:**
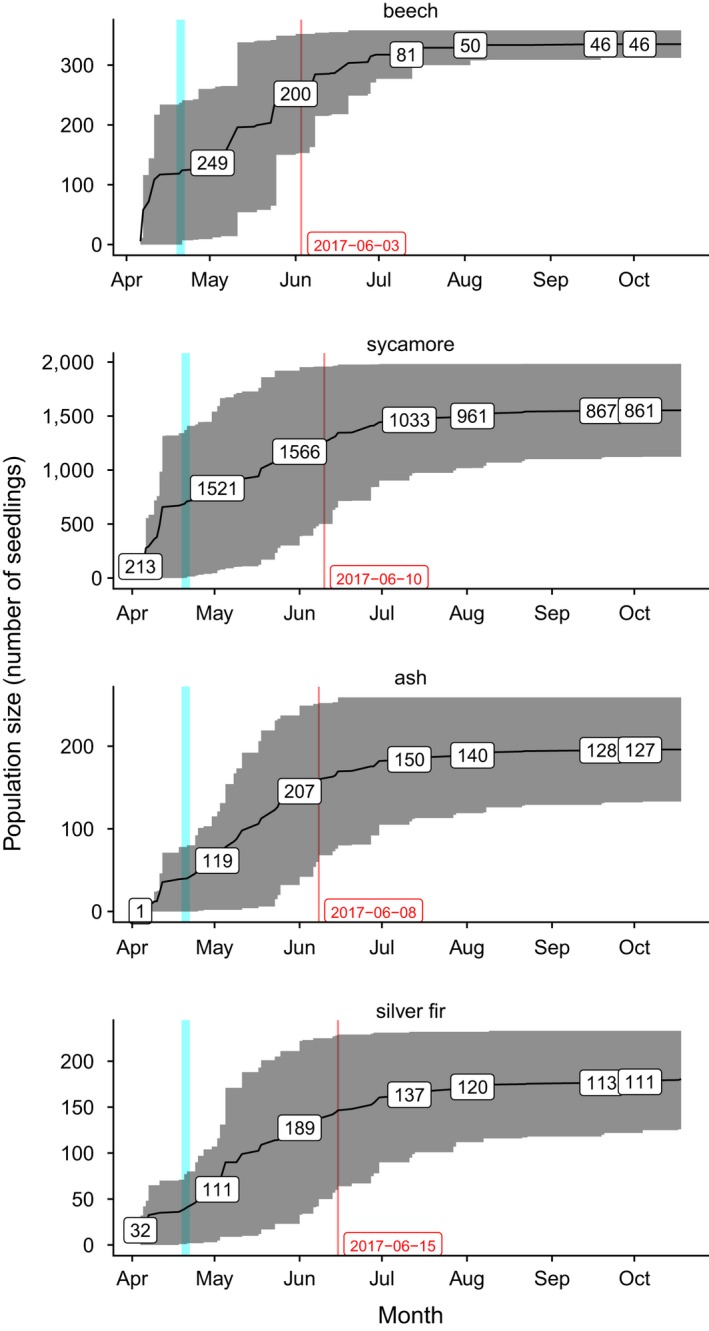
Seedling population size corresponding to the gray area, as difference between cumulative emergence events and mortality events (upper and lower curves, respectively), for the four most common species. Labels indicate the population size at the beginning of each month. Black line represents the running average population size. The vertical bar (cyan) indicates the frost events of mid‐April 2017. Vertical red lines represent the species‐specific time at which half of the mortality events of the first growing period occurred (cumulative mortality). Note that the scale of the y‐axis differs across species and that time in months is plotted on the x‐axis

We monitored 2,857 seedlings of ten tree species (Table 2). The most frequent species to emerge were *Acer pseudoplatanus* L. with 62% of all emerged seedlings, *Fagus sylvatica* L. with 13%, *Fraxinus excelsior* L. with 8%, and *Abies alba* Mill. with 8%. Other species were *Carpinus betulus* L., *Picea abies* (L.) H. Karst, *Acer platanoides* L., *Pinus sylvestris* L., *Quercus robur* L., and *Tilia cordata* Mill. Emergences at the Üetliberg site with 1,235 seedlings and at the Hönggerberg site with 1,066 seedlings were almost twice as high as at the Zürichberg site with 556 seedlings. The Zürichberg site differed not only regarding the total number of emergences, but also regarding the species frequencies: More than half (53.4%) of the emerged seedlings at the Zürichberg site were beech, while sycamore seedlings dominated the Üetliberg (76.6%) and Hönggerberg sites (67.9%). Emergence occurred mainly in spring (Figure 2): 56% of the seedlings emerged in March (i.e., they were present already in the first survey at the beginning of April 2017), 27% in April, 14% in May, and 2% in June. A few emergences were still observed in summer and fall, with 8 seedlings emerging on July, 4 in August, and one (sycamore) even in October, corresponding to 0.04% of the total emergence events. Four of these 13 late emergences were common oak seedlings.

**Table 2:** Overview of the emerged and surviving seedlings

Species and site	1st growing period				1st winter			2nd growing period		
	Emergence		Survival		Survival			Survival		
	No.	%	No.	% total	No.	% relative	% total	No.	% relative	% total
Total species*	2,857	100	1,048	36.7	898	85.7	31.4	662	73.7	23.2
Hönggerberg*	1,066	37.3	447	41.9	343	76.7	32.2	230	67.1	21.6
Üetliberg	1,235	43.2	468	37.9	433	92.5	35.1	338	78.1	27.4
Zürichberg	556	19.5	133	23.9	122	91.7	21.9	94	77.0	16.9
Sycamore*	1781	62.3	720	40.4	627	87.1	35.2	469	74.8	26.3
Hönggerberg*	724	40.7	324	44.8	252	77.8	34.8	168	66.7	23.2
Üetliberg	946	53.1	358	37.8	340	95.0	35.9	273	80.3	28.9
Zürichberg	111	6.2	38	34.2	35	92.1	31.5	28	80.0	25.2
Beech	358	12.5	47	13.1	40	85.1	11.2	29	72.5	8.1
Hönggerberg	32	8.9	6	18.8	4	66.7	12.5	2	50.0	6.3
Üetliberg	29	8.1	7	24.1	7	100.0	24.1	6	85.7	20.7
Zürichberg	297	83	34	11.4	29	85.3	9.8	21	72.4	7.1
Ash*	238	8.3	113	47.5	95	84.1	39.9	67	70.5	28.2
Hönggerberg*	118	49.6	62	52.5	47	75.8	39.8	36	76.6	30.5
Üetliberg	66	27.7	24	36.4	22	91.7	33.3	13	59.1	19.7
Zürichberg	54	22.7	27	50.0	26	96.3	48.1	18	69.2	33.3
Silver fir	233	8.2	105	45.1	96	91.4	41.2	72	75.0	30.9
Hönggerberg	67	28.8	21	31.3	18	85.7	26.9	11	61.1	16.4
Üetliberg	112	48.1	51	45.5	46	90.2	41.1	34	73.9	30.4
Zürichberg	54	23.2	33	61.1	32	97.0	59.3	27	84.4	50.0
Hornbeam*	143	5	23	16.1	16	69.6	11.2	10	62.5	7.0
Hönggerberg*	88	61.5	19	21.6	15	78.9	17.0	9	60.0	10.2
Üetliberg	37	25.9	4	10.8	1	25.0	2.7	1	100.0	2.7
Zürichberg	18	12.6	0	0.0						
Spruce	65	2.3	17	26.2	16	94.1	24.6	10	62.5	15.4
Hönggerberg	13	20	2	15.4	1	50.0	7.7	1	100.0	7.7
Üetliberg	31	47.7	15	48.4	15	100.0	48.4	9	60.0	29.0
Zürichberg	21	32.3	0	0.0						
Norway maple*	18	0.6	5	27.8	4	80.0	22.2	3	75.0	16.7
Hönggerberg*	13	72.2	3	23.1	2	66.7	15.4	1	50.0	7.7
Üetliberg	5	27.8	2	40.0	2	100.0	40.0	2	100.0	40.0
Zürichberg	0	0								
Pine	15	0.5	7	46.7	6	85.7	40.0	6	100.0	40.0
Hönggerberg	5	33.3	0	0.0						
Üetliberg	9	60	6	66.7	5	83.3	55.6	5	100.0	55.6
Zürichberg	1	6.7	1	100.0	1	100.0	100.0	1	100.0	100.0
Oak	5	0.2	5	100.0	4	80.0	80.0	2	50.0	40.0
Hönggerberg	5	100	5	100.0	4	80.0	80.0	2	50.0	40.0
Üetliberg	0	0								
Zürichberg	0	0								
Lime	1	0	1	100.0	0	0.0	0.0			
Hönggerberg	1	100	1	100.0	0	0.0	0.0			
Üetliberg	0	0								
Zürichberg	0	0								

Note

Total survival is defined as the percentage of emerged seedlings surviving until the end of October 2017, mid‐April 2018, and mid‐October 2018, respectively. Relative survival is defined as the percentage of seedlings surviving the period from early November 2017 to mid‐April 2018 (1st winter survival) and from mid‐April 2018 to mid‐October 2018 (2nd growing period survival), respectively.

* 202 seedlings from destroyed plots 13 and 14 at the Hönggerberg site are not listed (150 sycamores, 17 ashes, 30 hornbeams, and 5 Norway maples).

### 3.4 Survival during first growing period, winter and second growing period

After the first growing period, survival of all emerged seedlings across all ten species was 36.7%. The population size of seedling cohorts remained relatively stable during the first winter (survival 31.4%) and decreased only slightly during the second growing period (survival 23.2%) (Table 2). Half of all mortality events during the first growing period occurred by mid‐June (Figure 2). Across ash, beech, silver fir, and sycamore, 38% of the seedlings survived the first growing period (Table 2). The percentage of survival during the first growing period was much higher for ash (47.5%), silver fir (45.1%), and sycamore (40.4%) compared to beech (13.1%). The fraction of seedlings that survived the first growing period was higher at the Hönggerberg and Üetliberg sites (41.9% and 37.8%, respectively) than at the Zürichberg site (23.9%).

### 3.5 Survival differences within time‐constant variables

**Figure 3:**
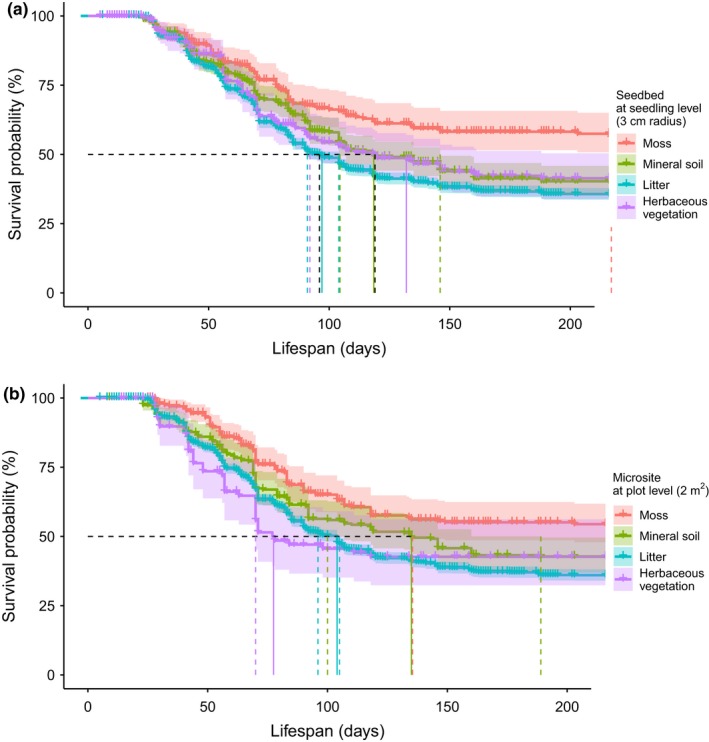
Kaplan–Meier survival curves estimate with 95% confidence intervals for seedbeds (at seedling level, 3 cm radius) and microsites (at plot level, 2 m^2^). The median survival time and 95% confidence intervals are indicated by the vertical solid line and dashed lines, respectively, which intersects the survival curve estimate at 50% probability (Equation 1). Short vertical lines indicate censored seedlings. Please note that here lifespan, not time as in Figure 2, is plotted on the x‐axis

Both seedbed and microsite on which seeds germinated were statistically related to seedling survival probabilities (Figure 3). At the end of the first growing period, that is, after 196 days, more than half of the seedlings were still alive on mossy substrate. In contrast, median survival time on both mineral soil and herbaceous vegetation (grasses and forbs) was 118 days (95% confidence intervals, CI, of 104–146 days and 86–161 days, respectively) and on litter 97 days (95% CI 91–104 days). The risk of death was higher for seedlings on both seedbeds and microsites covered by litter (84.7% and 68% higher hazard, respectively, based on Cox proportional hazards model), by herbaceous vegetation (53.0% and 65.6%, respectively), or by mineral soil (51.5% and 40.0%, respectively) than those covered by moss (*p* < 0.02; Table 3). Herbaceous vegetation (grasses and forbs) decreased survival in the first half of the growing period (survival did not decrease after ca. 110 days; Figure 3b).

**Table 3:** Fixed effects of seedbeds, microsites, species, sites, and month of emergence. Model output of Cox proportional hazards model (R function “coxph”)

Model	Factor level	Log‐hazard ratio (*LHR*)	Hazard ratio	Lower 95% CI	Upper 95% CI	*p*	Significance
Seedbed	Moss	Reference level					
	Mineral soil	0.415	1.515	1.171	1.959	0.002	***
	Litter	0.614	1.847	1.474	2.315	<0.001	***
	Herbaceous vegetation	0.426	1.530	1.124	2.084	0.007	**
Microsite	Moss	Reference level					
	Mineral soil	0.336	1.400	1.076	1.821	0.012	*
	Litter	0.519	1.680	1.362	2.072	<0.001	***
	Herbaceous vegetation	0.505	1.656	1.143	2.400	0.008	**
Species	Beech	Reference level					
	Sycamore	−0.763	0.466	0.411	0.529	<0.001	***
	Ash	−0.815	0.443	0.361	0.543	<0.001	***
	Silver fir	−0.803	0.448	0.365	0.551	<0.001	***
Site	Zürichberg	Reference level					
	Hönggerberg	−0.53741	0.584	0.5138	0.6644	<0.001	***
	Üetliberg	−0.29964	0.741	0.6544	0.8392	<0.001	***
Month of emergence	March	Reference level					
	April	0.654	1.923	1.725	2.144	<0.001	***
	May	0.975	2.652	2.301	3.056	<0.001	***

Note

Values and symbols are log‐hazard ratio (LHR, estimate, Equation 4), hazard ratio, lower and upper 95% confidence interval for the estimated hazard ratio, p‐values, from Wald tests of Cox models.

Significance classes for fixed effects are *p* > 0.05 (not significant), 0.05 ≥ *p *> 0.01 (weakly significant, *), 0.01 ≥ *p *> 0.001 (significant, **), 0.001 ≥ *p* (strongly significant, ***).

**Figure 4:**
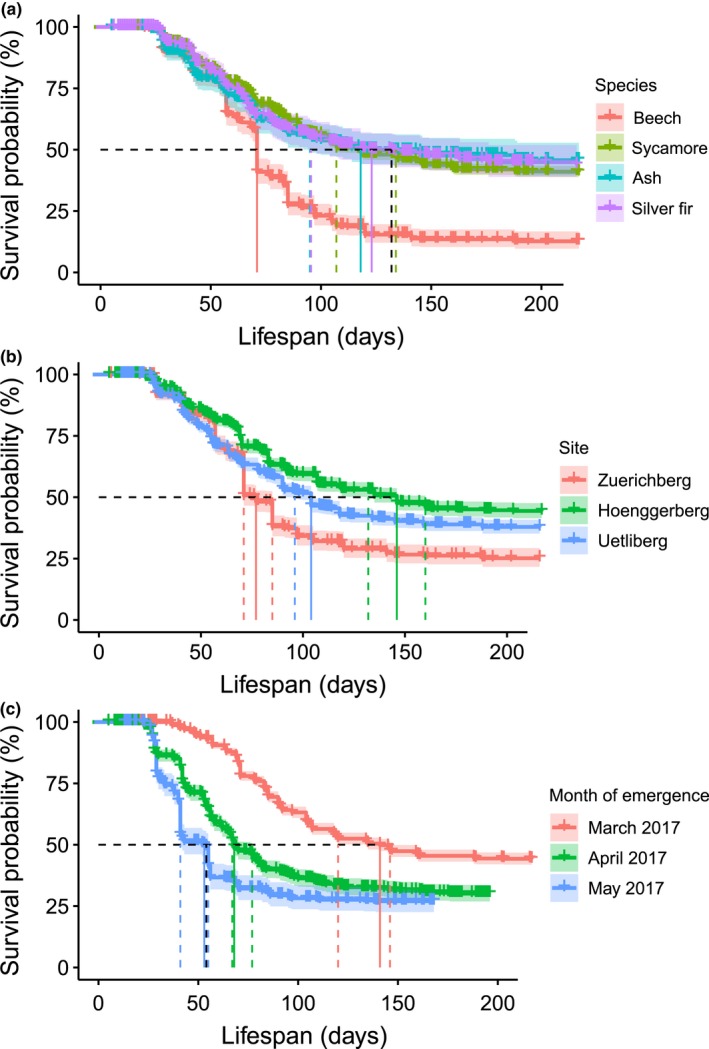
Kaplan–Meier survival curves estimate with 95% confidence intervals for species, sites and early versus late emergence time. The median survival time and 95% confidence intervals are indicated by the vertical solid line and dashed lines, respectively, which intersects the survival curve estimate at 50% probability (Equation 1). Short vertical lines indicate censored seedlings. Please note that here lifespan, not time as in Figure 2, is plotted on the *x*‐axis

The median survival time of beech (71 days) was significantly shorter than that of sycamore (119 days, 95% CI 118–135 days), silver fir (133 days, lower 95% confidence limit 95 days, upper confidence limit not defined), and ash (134 days, lower 95% confidence limit 110 days, upper confidence limit not defined) (Figure 4a). Compared to beech, the other three most common species had a significantly lower risk of death based on the Cox proportional hazards model: sycamore had a 53.4% lower hazard than beech (log‐hazard ratio LHR = −0.763, HR = *e^LHR^* = 0.466, that is, the hazard reduction is 0.534 or 53.4%, see page 74 of Moore (2016); *p* < 0.001, Table 3), ash a 55.7% lower hazard (LHR = −0.815, *p* < 0.001), and silver fir a 55.2% lower hazard (LHR = −0.803, *p* < 0.001). The high abundance of beech at Zürichberg contributed to the low median survival time of seedlings at that site, which was significantly lower (77 days, 95% CI 71–85 days) than at the Üetliberg site (104 days, 95% CI 98–108 days) and Hönggerberg site (146 days, 95% CI 132–160 days) (Figure 4b). With 25.9% and 41.6% lower hazards than at the Zürichberg, survival was significantly higher at Üetliberg and Hönggerberg (LHR = −0.300 and −0.537, respectively; both *p* < 0.001, Table 3).

Seedlings that emerged in March had a higher median survival time (141 days, 95% CI 120–146 days) than those that emerged in April (68 days, 95% CI 67–77 days) or May (53 days, 95% CI 41–55 days) (Figure 4c). The Cox model to test for the effect of emergence month on seedling survival indicated that seedlings with early emergence had higher survival probabilities (Table 3). The seedlings that emerged in April had a 92.3% higher hazard than those that emerged in March (LHR = 0.654, *p* < 0.001). Similarly, seedlings that emerged in May had a 165.2% higher hazard (LHR = 0.975, *p* < 0.001).

### 3.6 Survival models with time‐dependent covariates

#### 3.6.1 Seedling‐specific characteristics

As the timing of seedling emergence is species‐specific, separate random effects Cox models were fitted to each of the four most frequent species (Table 4). With each day that a seedling germinates later, the hazard increased by 1.6% for sycamore (LHR = 0.015, *p* < 0.001), 1.5% for beech (LHR = 0.015, *p* = 0.003), 1.2% for silver fir (LHR = 0.012, *p* = 0.007), and 0.7% for ash (LHR = 0.007, *p* = 0.140). In general, increasing seedling height and number of leaves decreased the hazard of death (Table 4). With each centimeter that a seedling was taller, the hazard decreased on average by 23.0% for beech (LHR = −0.261, *p* < 0.001), by 23.5% for sycamore (LHR = −0.268, *p* < 0.001), by 30.8% for ash (LHR = −0.368, *p* < 0.001), and by 41.5% for silver fir (LHR = −0.535, *p* < 0.001). Seedlings with the species‐specific typical number of cotyledons experienced lower mortality than those with a reduced number of cotyledons (resulting from browsing and other damages). An additional cotyledon decreased the hazard by 49.2% for sycamore (LHR = −0.677, *p* < 0.001), 38.3% for silver fir (LHR = −0.483, *p* < 0.001), 49.5% for ash (LHR = −0.683, *p* < 0.001), and 13.7% for beech (LHR = −0.148, *p* = 0.088). Similarly, an additional euphyll significantly reduced the hazard of death for the broadleaved species: by 21.3% for beech (LHR = −0.240, *p* < 0.001), 57.7% for sycamore (LHR = −0.860, *p* < 0.001), and 48.1% for ash (LHR = −0.655, *p* < 0.001).

**Table 4:** Fixed effects of emergence time (DOY), number of cotyledons, number of euphylls, seedling height (cm), negative minimum temperatures (°C), positive maximum temperatures (°C), mean daily precipitation sum (mm), and mean direct radiation (W/m^2^) for the species‐specific models of beech, sycamore, ash, and silver fir

Species‐specific model	Fixed effects	Log‐hazard ratio (*LHR*)	Hazard ratio	Lower 95% CI	Upper 95% CI	*p*	Significance
Beech	Emergence time	0.015	1.015	0.005	0.025	0.003	**
	Nr. cotyledons	−0.148	0.863	−0.317	0.022	0.088	
	Nr. euphylls	−0.240	0.787	−0.371	−0.109	<0.001	***
	Height	−0.261	0.770	−0.322	−0.200	<0.001	***
	Neg. min. temp	−0.408	0.665	−0.640	−0.176	0.001	***
	Pos. max. temp.	−0.007	0.993	−0.281	0.266	0.960	
	Mean precip.	−0.038	0.962	−0.103	0.026	0.240	
	Mean direct radiation	0.010	1.010	−0.004	0.025	0.170	
Sycamore	Emergence time	0.015	1.016	0.011	0.019	<0.001	***
	Nr. cotyledons	−0.677	0.508	−0.835	−0.519	<0.001	***
	Nr. euphylls	−0.860	0.423	−0.964	−0.755	<0.001	***
	Height	−0.268	0.765	−0.302	−0.233	<0.001	***
	Neg. min. temp	−0.002	0.998	−0.121	0.117	0.980	
	Pos. max. temp.	0.006	1.006	−0.126	0.139	0.920	
	Mean precip.	−0.027	0.973	−0.064	0.010	0.150	
	Mean direct radiation	0.006	1.006	−0.004	0.015	0.240	
Ash	Emergence time	0.007	1.007	−0.003	0.023	0.140	
	Nr. cotyledons	−0.683	0.505	−1.039	−0.327	<0.001	***
	Nr. euphylls	−0.655	0.519	−0.983	−0.327	<0.001	***
	Height	−0.368	0.692	−0.504	−0.233	<0.001	***
	Neg. min. temp	−0.476	0.621	−1.027	0.074	0.090	
	Pos. max. temp.	0.005	1.005	−0.299	0.309	0.970	
	Mean precip.	−0.036	0.965	−0.148	0.076	0.530	
	Mean direct radiation	0.008	1.008	−0.016	0.032	0.500	
Silver fir	Emergence time	0.012	1.012	0.003	0.020	0.007	**
	Nr. cotyledons	−0.483	0.617	−0.654	−0.312	<0.001	***
	Nr. euphylls	−0.217	0.805	−0.493	0.059	0.120	
	Height	−0.535	0.585	−0.673	−0.398	<0.001	***
	Neg. min. temp	−0.067	0.935	−0.456	0.321	0.730	
	Pos. max. temp.	−0.453	0.636	−0.907	0.001	0.051	
	Mean precip.	−0.032	0.968	−0.144	0.080	0.570	
	Mean direct radiation	0.006	1.006	−0.010	0.022	0.460	

Note

Model output of mixed‐effects Cox proportional hazards model (R function “coxme”). Values and symbols are log‐hazard ratio (LHR, estimate, Equation 4), hazard ratio, lower and upper 95% confidence limit for the estimated hazard ratio, *p*‐values, from Wald tests of Cox models.

Significance classes for fixed effects are *p* > 0.05 (not significant), 0.05 ≥ *p *> 0.01 (weakly significant, *), 0.01 ≥ *p *> 0.001 (significant, **), 0.001 ≥ *p* (strongly significant, ***).

#### 3.6.2 Abiotic factors

An increase by 1°C of negative minimum temperatures (i.e., less severe frost) reduced the hazard of death for beech by 33.5% (*p* = 0.001) and ash by 37.9% (*p* = 0.090), that is, the colder, the higher the hazard and thus the lower the survival probability (Table 4), which is clearly evident from the steep increase of the mortality curve for beech in May as a consequence of the April frost events (Figure 2). Silver fir profited from high maximum temperatures, as the effect was marginally significant with a 1°C increase in positive maximum temperatures reducing the hazard of death by 36.4% (*p* = 0.051). This effect was not significant for the other species. A 1 mm increase in the mean daily precipitation sum within a survey interval did not significantly influence seedling survival (species‐specific P‐values ranged from 0.570 to 0.150), but still a slightly negative effect on the hazard was evident (Table 4): the more precipitation and thus the more humid the soil (commensurate to the drought period in May and June 2017, see section Weather), the lower was the hazard of death. Thus, the low amounts of May/June precipitation did not significantly influence seedling survival. A 1 W/m^2^ increase in light availability did not significantly affect seedling survival (Table 4). The mean daily effective direct radiation was slightly positively correlated with the hazard of death for the four most common species.

## 4 DISCUSSION

In this study, the development of tree seedling cohorts during the first growing period showed that nearly two‐thirds of the seedlings died. Median survival times and survival curves varied strongly among species, with beech having the lowest survival, caused most likely by frost damages. Our results also highlight the fundamental importance of emergence time for survival, showing that early‐emerging seedlings experienced lower mortality during the first growing period in spite of having faced severe late frost events. Furthermore, our findings indicate that seedbed and microsite as well as seedling characteristics and, to a minor extent, abiotic factors influenced seedling survival.

### 4.1 Effects of emergence time on seedling survival

The timing of emergence strongly influences seedling survival. Along the gradient of emergence time, we found that early emergence reduces the hazard of death. Our finding agrees with the meta‐analysis by Verdú and Traveset (2005). In our study, when considering the trade‐off between early emergence being bound to higher frost risk and late emergence being bound to a shorter growing period, the former appears to be more advantageous for seedling survival of the studied tree species. The smaller size both above and below ground, resulting from shorter growing period due to late emergence, may be problematic during drought spells, as smaller rooting systems fail to absorb sufficient quantities of water. Thus, the length of the growing period appears to be decisive for seedling survival, especially for seedlings of succeeding type phenology (i.e., those with continued vertical elongation and leaf production during the growing period, such as beech), which can grow longer in a longer growing period.

Early‐emerging seedlings feature higher growth rates than those emerging later (de Luis, Verdú, & Raventós, 2008; Orrock & Christopher, 2010; Trimble & Tryon, 1969), which may be due to the fact that early emergence implies exposure to higher light availability and thus higher carbon gain (Augspurger & Bartlett, 2003), with photosynthetic activity peaking before canopy closure (Augspurger, Cheeseman, & Salk, 2005). Indeed, higher light availability prior to the development of the forest canopy is another advantage that early‐emerging seedlings benefit from (Winkler, Hülber, & Hietz, 2005). Thus, in spite of the risk of late frost events, emerging early in the growing period is beneficial for tree seedling survival in beech‐dominated forests. These results are in line with a study on survival of *Acer rubrum* seedlings, where a delay of three weeks in the emergence time reduced survival probability from 80% to 20% (Jones et al., 1997). Similar patterns were observed for *Pinus sylvestris* seedlings, with time of emergence being decisive for survival across different microsites (Castro, 2006). The difference in survival probability due to the temporal delay in emergence can last until the sapling stage (Streng, Glitzenstein, & Harcombe, 1989).

The timing of seedling emergence is determined by both abiotic and biotic factors. Examples of the formers are water and temperature, with adequate moisture supply and warmth accelerating seedling emergence by inducing seed imbibition (i.e., water uptake by the germinating seed) and increasing the speed of germination, whereas cold spells and drought postponing seedling emergence (Farmer, 1997). Further, an example of biotic factors is the number of viable seeds present in the forest floor together with the presence of several intra‐ and interspecific individuals in the understorey, which may generate competitive conditions (Leverett, 2017), possibly leading to premature (Dyer, Fenech, & Rice, 2000) or delayed emergence times (Inouye, 1980; Leverett et al., 2018; Seiwa, 2000). Emerging late in the growing period may be either a species‐specific feature or a bet‐hedging strategy. On the one hand, delayed emergence may be advantageous for warmth‐demanding tree species like hornbeam and oak, as shown by the four oak seedlings emerging in late summer. On the other hand, delayed emergence may represent a biological strategy in the event that the majority of the early‐emerging population is not successful (Gremer & Venable, 2014; Mathias & Kisdi, 2002).

### 4.2 Effects of seedling characteristics on seedling survival

The observed decline in mortality risk with increasing seedling height agrees with observations that initial height is positively correlated with survival probability (Fidej, Rozman, & Diaci, 2018; Oshima, Tokumoto, & Nakagawa, 2014). We used seedling height as a time‐varying variable instead of initial height, because of the uncertainty related to double censored emergence times. Beside emergence time, seed size and maternal or genetic effects may cause differences in seedling height (Kitajima & Fenner, 2000). Seedlings of the flush type phenology (i.e., shoot elongation and annual leaf production are completed at the time of emergence, such as in silver fir or Norway spruce) are usually much shorter than seedlings of the succeeding type phenology (Orman, Adamus, & Szewczyk, 2016). Seed size is decisive for the growth of seedlings of the flush type phenology, whereas emergence time is important for the growth of the succeeding type phenology (Seiwa, 2000). Early emergence contributes not only to increased seedling height, but also to elevated biomass and stem diameter (Boyer, Duba, & South, 1987; Orrock & Christopher, 2010). In addition, an increasing number of leaves was associated with higher survival probability, most likely because these seedlings are more vigorous and have a larger leaf area (Yi, Bartlow, Curtis, Agosta, & Steele, 2019).

### 4.3 Effects of substrate on seedling survival

We found seedbeds and microsites covered by moss to be most beneficial for seedling survival. Their high moisture retention capacity may have mitigated mortality (Battaglia & Reid, 1993), particularly during slight drought. The observed switch from a competitive to a rather supportive role of herbaceous vegetation may be due to the fact that leaf unfolding of herbaceous vegetation (including annual grasses and forbs) at the forest floor occurred concurrently to those of canopy trees, starting in mid‐April 2017 only. Thus, approximately half of the seedlings emerged before both events and profited from both low competition and high light availability at the forest floor. When light availability at the forest floor begins to decrease, competition between tree seedlings and herbaceous vegetation increases, as both suffer from the darker environment. Herbaceous vegetation is then less competitive in the second half of the growing period, for example, *Allium ursinum* L. at the Üetliberg site. This ambiguous role of herbaceous vegetation has already been found in other studies (Maher, Germino, & Hasselquist, 2005; Royo & Carson, 2008) and has been suggested to change with seedling age and seasons (Loranger, Zotz, & Bader, 2017). In addition to the evidence that different seedbeds and microsites influence seedling survival, we do not rule out interactions between the surrounding overstorey structure or understorey vegetation (Shen & Nelson, 2018) and other abiotic factors such as temperature, precipitation, and light availability (Lucas‐Borja et al., 2016).

### 4.4 Effects of abiotic factors on seedling survival

Under climate change, late frost events in spring may become more common due to the advancing spring phenology, thus the risk of frost damage is likely to increase (Augspurger, 2013; Bigler & Bugmann, 2018). In our study, more severe frost significantly increased the hazard of death only for beech, and for ash, the effect was marginally significant (Table 4). The frost susceptibility of beech caused survival to be particularly low (13.1%) compared to the other species. However, low survival seems to be genus‐specific for beech, with similar percentages as in our study being observed for *F. crenata* Blume (Akashi, 1997) and *F. grandifolia* var. *mexicana* (Alvarez‐Aquino & Williams‐Linera, 2002; Houle, 1994). Sycamore, ash, and silver fir featured moderate survival during the first growing period, ranging from 40% to 50%, similar to findings for other seedlings of these genera (Gardescu, 2003; Jinks, Willoughby, & Baker, 2006; Kellman, 2004; Macmillan & Aarssen, 2017). Although the negative temperatures during the severe late frost event in April 2017 did not reach the species‐ and age‐specific temperatures that are lethal for 50% of the seedling population (Hofmann et al., 2014), we observed frost‐induced damages as well as mortality. A few seedlings were able to survive even with serious damages by producing new euphylls. While there is no information on this response from seedling studies, refoliation based on the activation of dormant buds has been observed in saplings and adult trees (Augspurger, 2009). Frost damages occurred especially in canopy gaps, as also observed by Li et al. (2018), because a tall canopy, even if not in full leaf status yet, helps to mitigate frost injury and avoid death (Negi, Negi, & Singh, 1996). Under open conditions, frost may cause higher mortality and more serious damages to seedlings than under a forest canopy, and therefore, late emergence may be advantageous for seedlings under these conditions. Thus, frost‐induced seedling mortality is a species‐specific phenomenon occurring at a small spatial scale.

Our results showed that increasing positive maximum temperatures decreased the hazard of death for silver fir, whereas the other species were indifferent. Silver fir mainly emerged in small patches of conifers, where it may have been shielded from the highest heat spells, which in turn may explain why this species profited from warmth. Summer drought often causes seedling mortality (Kolb & Robberecht, 1996; McDowell et al., 2008). In spite of the exceptionally dry period in late spring/early summer 2017, we did not find significant evidence that precipitation mitigates the hazard, possibly because we did not measure soil moisture continuously or the local climate station measurements do not reflect the effective rainfall at our sites. Hence, we could not confirm the expected declining survival due to the concurring effect of heat and drought as found by for example, Way, Crawley, and Sage (2013).

Although light availability is fundamental for photosynthetic activity (Lin et al., 2014; Mason, Edwards, & Hale, 2004), we found only weak and nonsignificant effects on the hazard of death. This weak dependence of seedling survival on light has already been observed (Harmer, 1999; Szwagrzyk, Szewczyk, & Bodziarczyk, 2001). However, an excessive exposition to direct radiation, combined with low water content in soil, may reduce seedling survival.

## 5 CONCLUSIONS

2017 was very favorable for the purposes of this study because both a severe late frost and summer drought occurred in the same year. However, the results of 2017 cannot be extrapolated to other years. However, our study on the development of germination and seedling survival in 2017 provides empirical evidence of the fundamental ecological importance of emergence time in combination with late frost events for seedling survival of multiple tree species in temperate forests of Central Europe. The timing of seedling emergence represents a trade‐off between decreasing the frost risk and increasing the length of the growing period, which has not been systematically investigated before. While early‐emerging seedlings had high survival probabilities in spite of facing an exceptionally severe spring frost event, survival decreased with later emergence time. Our results further indicate seedling‐ and substrate‐specific effects such as the number of leaves and height along with seedbed and microsite as decisive for survival during the first growing period. Apart from the adverse effect of frost on the survival of beech seedlings, climatic conditions and light availability had relatively weak impacts on seedling survival. Our findings nevertheless indicate that tree seedling survival during the first growing period depends on a multitude of interacting biotic and abiotic factors. In light of the relatively low survival during the first growing period, the first months of a seedling's life appear to represent a major bottleneck for successful tree regeneration, which will ultimately have an impact on the future development of forest stands.

## CONFLICT OF INTEREST

The authors declare no competing interests.

## AUTHOR'S CONTRIBUTIONS

E.B., C.B. and H.B. conceived the ideas and designed the methodology; E.B. collected the data; E.B. and C.B. analyzed the data; E.B. led the writing of the manuscript. All authors contributed critically to the drafts and gave final approval for publication.

## Data Availability

Data available from the Dryad Digital Repository: https://doi.org/10.5061/dryad.7h95044
